# Supplementary material for: Evaluation of the effectiveness of combined staged surgical treatment in patients with keratoconus
Source: PLoS One. 2022 Mar 7;17(3):e0264030. doi: 10.1371/journal.pone.0264030 (PMC8901062; doi:10.1371/journal.pone.0264030)
Supplement: Attachment — Submitted filename: PONE-D-21-31417-converted.docx [file pone.0264030.s001.docx]

PLOS ONE

**Evaluation of the effectiveness of combined staged surgical treatment in patients with keratoconus**

--Manuscript Draft--

| **Manuscript Number:** | PONE-D-21-31417 |
| --- | --- |
| **Article Type:** | Research Article |
| **Full Title:** | Evaluation of the effectiveness of combined staged surgical treatment in patients with keratoconus |
| **Short Title:** | Combined staged surgical treatment in keratoconus |
| **Corresponding Author:** | Polad Maharramov  : National Centre of Ophthalmology Baku, AZERBAIJAN |
| **Keywords:** | corneal collagen cross-linking; keratoconus; intrastromal corneal ring segments; topography-guided photorefractive keratectomy |
| **Abstract:** | Purpose  This study performs comparative assessment of the results of different types of two- stage surgical treatment in patients with keratoconus, including combination of corneal collagen cross-linking with intrastromal corneal ring segments followed by topography- guided photorefractive keratectomy.  Materials and methods  Prospective review of 101 patients (101 eyes) with keratoconus was performed. Patients underwent corneal collagen cross-linking (32 patients), intrastromal corneal ring segments (48 patients), and a combination of these two procedures (21 patients). Transepithelial topography-guided photorefractive keratectomy as the second stage of treatment was performed after 8 months in all patients. Main outcome measures were visual acuity (uncorrected distance and corrected distance) and corneal topographic indices.  Results  Comparison of the studied parameters after first stage surgical treatment between non- combined and combined groups demonstrated a significant statistical difference only for uncorrected distance visual acuity values (0.21 ± 0.04; 0.45 ± 0.03 and 0.51 ± 0.04; p<0.05). We noticed significant improvement of visual acuity and key corneal topographic indices after topography-guided photorefractive keratectomy in all studied groups (p<0.05). According to some indices after second stage surgical treatment (uncorrected distance visual acuity, cylindrical refraction, spherical equivalent and index of vertical asymmetry) results were statistically significantly better in combined group than in non-combined groups (p<0.05).  Conclusions  This study shows that combined two-stage surgical treatment of keratoconus, consisting of intrastromal corneal ring segments with corneal collagen cross-linking followed by topography-guided photorefractive keratectomy, is clinically more effective than the use of non-combined two-stage techniques. |
| **Order of Authors:** | Polad Maharramov |
|  | Fidan A. Aghayeva |
| **Additional Information:** |  |
| **Question** | **Response** |
| **Financial Disclosure** | The authors received no specific funding for this work. |

| Enter a financial disclosure statement that describes the sources of funding for the work included in this submission. Review the [submission guidelines](http://journals.plos.org/plosone/s/submission-guidelines#loc-financial-disclosure-statement) for detailed requirements. View published research articles from [*PLOS ONE*](http://journals.plos.org/plosone/) for specific examples.  This statement is required for submission and **will appear in the published article** if the submission is accepted. Please make sure it is accurate. |  |
| --- | --- |
| **Unfunded studies**  Enter: *The author(s) received no specific funding for this work.*  **Funded studies**  Enter a statement with the following details:   - Initials of the authors who received each award - Grant numbers awarded to each author - The full name of each funder - URL of each funder website - Did the sponsors or funders play any role in the study design, data collection and analysis, decision to publish, or preparation of the manuscript? - **NO** - Include this sentence at the end of your statement: *The funders had no role in study design, data collection and analysis, decision to publish, or preparation of the manuscript.* - **YES** - Specify the role(s) played. |  |
| * typeset |  |
| **Competing Interests**  Use the instructions below to enter a competing interest statement for this submission. On behalf of all authors, disclose any [competing interests](http://journals.plos.org/plosone/s/competing-interests) that could be perceived to bias this work—acknowledging all financial support and any other relevant financial or non- financial competing interests.  This statement is required for submission and **will appear in the published article** if | The authors have declared that no competing interests exist. |

| the submission is accepted. Please make sure it is accurate and that any funding sources listed in your Funding Information later in the submission form are also declared in your Financial Disclosure statement.  View published research articles from  [*PLOS ONE*](http://journals.plos.org/plosone/) for specific examples. |  |
| --- | --- |
| **NO authors have competing interests**  Enter: *The authors have declared that no competing interests exist.*  **Authors with competing interests**  Enter competing interest details beginning with this statement:  *I have read the journal's policy and the authors of this manuscript have the following competing interests: [insert competing interests here]* |  |
| * typeset |  |
| **Ethics Statement**  Enter an ethics statement for this submission. This statement is required if the study involved:   - Human participants - Human specimens or tissue - Vertebrate animals or cephalopods - Vertebrate embryos or tissues - Field research   Write "N/A" if the submission does not require an ethics statement.  General guidance is provided below. Consult the [submission guidelines](http://journals.plos.org/plosone/s/submission-guidelines#loc-guidelines-for-specific-study-types) for detailed instructions. **Make sure that all information entered here is included in the Methods section of the manuscript.** | The study adhered to the tenets of the Declaration of Helsinki and was approved by the Ethics Committee of the National Centre of Ophthalmology named after academician Zarifa Aliyeva, Azerbaijan. The written consent to participate was obtained from all participants. |

| **Format for specific study types**  **Human Subject Research (involving human participants and/or tissue)**   - Give the name of the institutional review board or ethics committee that approved the study - Include the approval number and/or a statement indicating approval of this research - Indicate the form of consent obtained (written/oral) or the reason that consent was not obtained (e.g. the data were analyzed anonymously)   **Animal Research (involving vertebrate animals, embryos or tissues)**   - Provide the name of the Institutional Animal Care and Use Committee (IACUC) or other relevant ethics board that reviewed the study protocol, and indicate whether they approved this research or granted a formal waiver of ethical approval - Include an approval number if one was obtained - If the study involved *non-human primates*,   add *additional details* about animal welfare and steps taken to ameliorate suffering   - If anesthesia, euthanasia, or any kind of animal sacrifice is part of the study, include briefly which substances and/or methods were applied   **Field Research**  Include the following details if this study involves the collection of plant, animal, or other materials from a natural setting:   - Field permit number - Name of the institution or relevant body that granted permission |  |
| --- | --- |
|  |  |
| **Data Availability**  Authors are required to make all data underlying the findings described fully available, without restriction, and from the time of publication. PLOS allows rare exceptions to address legal and ethical concerns. See the [PLOS Data Policy](http://journals.plos.org/plosone/s/data-availability) and [FAQ](http://journals.plos.org/plosone/s/data-availability#loc-faqs-for-data-policy) for detailed information. | Yes - all data are fully available without restriction |

| A Data Availability Statement describing where the data can be found is required at submission. Your answers to this question constitute the Data Availability Statement and **will be published in the article**, if accepted. |  |
| --- | --- |
| **Important:** Stating ‘data available on request from the author’ is not sufficient. If your data are only available upon request, select ‘No’ for the first question and explain your exceptional situation in the text box. |  |
| Do the authors confirm that all data underlying the findings described in their manuscript are fully available without restriction? |  |
| **Describe where the data may be found in full sentences. If you are copying our sample text, replace any instances of XXX with the appropriate details.**   - If the data are **held or will be held in a public repository**, include URLs, accession numbers or DOIs. If this information will only be available after acceptance, indicate this by ticking the box below. For example: *All XXX files are available from the XXX database (accession number(s) XXX, XXX.*). - If the data are all contained **within the manuscript and/or Supporting Information files**, enter the following: *All relevant data are within the manuscript and its Supporting Information files.* - If neither of these applies but you are able to provide **details of access elsewhere**, with or without limitations, please do so. For example:   *Data cannot be shared publicly because of [XXX]. Data are available from the XXX Institutional Data Access / Ethics Committee (contact via XXX) for researchers who meet the criteria for access to confidential data.*  *The data underlying the results presented in the study are available from (include the name of the third party* | All relevant data are within the manuscript and its Supporting Information files. |

| *and contact information or URL).*   - This text is appropriate if the data are owned by a third party and authors do not have permission to share the data.   * typeset |  |
| --- | --- |
| Additional data availability information: |  |

Manuscript	[Click here to access/download;Manuscript;Manuscript.docx](https://www.editorialmanager.com/pone/download.aspx?id=29920601&guid=2f8a5380-bb3b-4385-9b47-ec2d898333a9&scheme=1)

# 1 Evaluation of the effectiveness of combined staged surgical treatment in patients with

2 **keratoconus**
3 Polad M. Maharramov^1^, Fidan A. Aghayeva^1^
4 ^1^National Centre of Ophthalmology named after academician Zarifa Aliyeva, Baku, Azerbaijan 5

6

7

8

9

10

# 11 Corresponding author:

12 Polad M. Maharramov, PhD
13 Department of Cornea,
14 National Centre of Ophthalmology named after academician Zarifa Aliyeva, Baku, Azerbaijan
15 Javadkhan street 32/15, AZ 1114 Baku

16	Phone: +994503173082

17	Fax: +994124928008

18	Email: [maharramov@mail.ru](mailto:maharramov@mail.ru)

19	ORCID: 0000-0002-7211-0343

20

21

22

23

24

25

## 26 ABSTRACT

27 ***Purpose*** – This study performs comparative assessment of the results of different types of two-
28 stage surgical treatment in patients with keratoconus, including combination of corneal
29 collagen cross-linking with intrastromal corneal ring segments followed by topography-guided
30 photorefractive keratectomy.
31 ***Materials and methods*** – Prospective review of 101 patients (101 eyes) with keratoconus was
32 performed. Patients underwent corneal collagen cross-linking (32 patients), intrastromal
33 corneal ring segments (48 patients), and a combination of these two procedures (21 patients).
34 Transepithelial topography-guided photorefractive keratectomy as the second stage of
35 treatment was performed after 8 months in all patients. Main outcome measures were visual
36 acuity (uncorrected distance and corrected distance) and corneal topographic indices.
37 ***Results*** – Comparison of the studied parameters after first stage surgical treatment between
38 non-combined and combined groups demonstrated a significant statistical difference only for
39 uncorrected distance visual acuity values (0.21 ± 0.04; 0.45 ± 0.03 and 0.51 ± 0.04; p<0.05).
40 We noticed significant improvement of visual acuity and key corneal topographic indices after
41 topography-guided photorefractive keratectomy in all studied groups (p<0.05). According to
42 some indices after second stage surgical treatment (uncorrected distance visual acuity,
43 cylindrical refraction, spherical equivalent and index of vertical asymmetry) results were
44 statistically significantly better in combined group than in non-combined groups (p<0.05).
45 ***Conclusions*** – This study shows that combined two-stage surgical treatment of keratoconus,
46 consisting of intrastromal corneal ring segments with corneal collagen cross-linking followed
47 by topography-guided photorefractive keratectomy, is clinically more effective than the use of
48 non-combined two-stage techniques.
49 ***KEYWORDS:*** corneal collagen cross-linking; keratoconus; intrastromal corneal ring
50 segments; topography-guided photorefractive keratectomy

## 51 INTRODUCTION

52 Keratoconus is a progressive bilateral pathology of cornea, which is manifested by its thinning
53 and protrusion that lead to astigmatism and deterioration of vision [1]. Spectacles and contact
54 lenses, corneal	collagen	cross-linking (CXL)	with	photorefractive keratectomy (PRK),
55 intrastromal corneal ring segments (ICRS), keratoplasty (penetrating and deep anterior
56 lamellar), and combinations of the above-mentioned methods could be used in the treatment of
57 keratoconus, depending on its severity and grade. All treatment methods are believed to be
58 effective and safe. Such conclusion is often based on the results of comparison of visual acuity,
59 refraction and corneal topographic indices before and after surgery. Egyptian scientists
60 presented data on the efficacy of combination of PRK with CXL [2]. There were several reports
61 about the combination of CXL, ICRS and topography-guided PRK (TG-PRK). The authors
62 consider several options for the combination of different treatment methods [5]. It is believed
63 that corneal surgery in keratoconus should both provide an improvement in vision and also
64 prevent the progression of pathology [4]. An analytical review of literature indicates that the
65 use of only CXL for the treatment of keratoconus has almost no effect on functional indicators,
66 while effectively stops the progression of pathology due to an increase in corneal strength [5].
67 The combination of CXL with PRK has a double effect: a significant improvement in visual
68 acuity and an increase in corneal stability. A reported conclusion about the clinical efficacy of
69 the combination of ICRS with CXL in the treatment of keratoconus was based on the
70 comparison of pre- and postoperative indicators of visual acuity, topographic indices, and
71 corneal thickness [6]. A more extended and detailed review of the protocols for the combined
72 use of CXL (CXL plus) in combination with PRK, phototherapeutic keratectomy (PTK), ICRS,
73 and implantation of phakic intraocular lenses demonstrates that further studies are required to
74 determine the most effective treatment strategy [7]. Therefore, we decided to compare the
75 results of different types of two-stage surgical treatment in patients with keratoconus, including
76 combination of CXL with ICRS followed by TG-PRK.

## 77 MATERIALS AND METHODS

78 We performed a prospective clinical study including 101 patients (101 eyes) with keratoconus
79 who underwent two-stage surgical management at the National Centre of Ophthalmology
80 named after academician Zarifa Aliyeva, Azerbaijan. Mean age of the patients was 20.1±8.4
81 years, 53 were males and 48 females. The occupations of included patients were office workers
82 (42 patients), oil workers (20 patients), doctors (15 patients), and other (24 patients). The study
83 adhered to the tenets of the Declaration of Helsinki and was approved by the Ethics Committee
84 of the National Centre of Ophthalmology named after academician Zarifa Aliyeva, Azerbaijan.
85 The written consent to participate was obtained from all participants.
86 At the first stage of treatment, from January 2017 to January 2018, 32 patients (32 eyes)
87 underwent only CXL (CXL group), 48 patients (48 eyes) underwent only ICRS (ICRS group)
88 and 21 patients with keratoconus stage II – III, according to Amsler-Krumeich classification,
89 underwent a combination of CXL and ICRS (first ICRS, after 24 hours CXL, ICRS+CXL
90 group). All patients underwent transepithelial TG-PRK after 8 months as the second stage of
91 treatment.
92 Surgical technique was standard. KeraRing (Mediphacos, Belo Horizonte, Brazil) was used for
93 ICRS, intracorneal tunnel with the inside and outside diameters 4.4 mm and 5-6 mm,
94 respectively, was created by using femtosecond laser Alcon WaveLight® FS200 (USA). The
95 ring was implanted in the thinnest part of the cornea at a depth of 90 μm from the endothelium

96	(Fig 1).

97 **Fig 1. Visualization of ICRS.** ICRS = intrastromal corneal ring segments.
98 To perform CXL, corneal de-epithelialization was carried out in a treatment zone of 7 mm
99 under topical anaesthesia, then 0.1% riboflavin solution (Riboflavin Medio Cross) was applied
100 on the cornea every 5 minutes for 30 minutes and exposure to UVA in six steps of 5 minutes
101 was performed. Contact lens was applied at the end of surgery. Transepithelial TG-PRK was
102 performed 8 months later after obtaining stable refractive results: 50 μm thick corneal
103 epithelium was removed by PTK under topical anaesthesia, then ablation of the stroma was
104 performed by PRK.
105 All patients were examined before surgery and 1, 3, 6, 8 months after every surgical procedure
106 during first and second stage treatment (follow-up of patients in all groups was more than 16
107 months). The examination included measurement of uncorrected distance and corrected
108 distance visual acuity (UDVA and CDVA), autorefractometry (TOMEY RC-5000, Japan),
109 noncontact tonometry (TOMEY FT-1000, Japan), biomicroscopy (Tomey TSL 5000, Tomey,
110 Japan), Pentacam corneal tomography (Oculus Wetzlar, Germany), Wavelight Oculyzer
111 (ALCON, USA), WaveLight*®* Topolyzer® VARIO (ALCON, USA), anterior segment OCT
112 (Cirrus HD-OCT 5000, Zeiss, Germany), ultrasound pachymetry, and funduscopy.
113 Statistical analysis was conducted using analysis methods of quantitative and qualitative
114 characteristics [8] with Microsoft Excel-2010.

## 115 RESULTS

116 Data on vision acuity and corneal topography before surgery and 6 months after CXL, ICRS
117 and ICRS with CXL are shown in Table 1. These data indicate that visual acuity and corneal
118 topographic indices changed noticeably at 6 months after all three types of first stage surgical
119 treatment, thus confirming the achievement of the expected effect. The clinical results after
120 combination of CXL and ICRS are comparatively better than in non-combined groups.
121 Although, a statistically significant difference between combined and non-combined groups
122 was revealed only for UDVA value (p <0.05). All operated patients underwent TG-PRK 8
123 months after first stage treatment. Clinical parameters before TG-PRK and 6 months after ТG-
124 PRK are given in Table 2.

**Table 1. Clinical parameters before and 6 months after CXL, ICRS and ICRS + CXL**

Indices	CXL	ICRS	ICRS + CXL

| before | after | before | after | before | after |
| --- | --- | --- | --- | --- | --- |
| 0.15±0.03 | 0.21±0.04* | 0.13±0.03 | 0.45±0.03* | 0.12±0.03 | 0.51±0.04*^ |
| 0.36±0.04 | 0.42±0.061* | 0.38±0.03 | 0.79±0.03* | 0.31±0.04 | 0.89±0.03* |
| -5.20±0.12 | -4.80±0.13 | -5.36±0.14 | -2.06±0.14 | -6.22±0.13 | -1.54±0.14 |
| -2.11±0.12 | -1.89±0.10 | -6.04±0.11 | -1.88±0.10 | -7.08±0.11 | -1.87±0.10 |
| -5.61±0.14 | 5.10±0.11* | -5.50±0.11 | 5.18±0.14* | -5.58±0.11 | 5.08±0.12* |
| 48.90±0.91 | 47.11±0.12* | 50.81±0.12 | 44.32±0.11* | 50.78±0.10 | 43.11±0.12 |
| -7.65±0.08 | -7.78±0.07 | -7.68±0.09 | -7.80±0.07 | -7.65±0.08 | 7.80±0.07 |
| 456±4.1 | 448±3.8 | 458±4.5 | 445±4.2 | 459±4.1 | 446±4.0 |
| 57.0±0.2 | 56.1±0.3* | 56.9±0.3 | 55.8±0.2* | 56.8±0.3 | 55.4±0.2* |
| -0.85±0.05 | -0.89±0.06 | -0.84±0.05 | -0.88±0.04 | -0.85±0.04 | -0.88±0.04 |
| 2.26±0.05 | 2.49±0.06* | 2.27±0.05 | 2.50±0.06* | 2.26±0.05 | 2.51±0.07* |
| 4.85±0.21 | 4.34±0.21 | 4.86±0.22 | 4.35±0.22 | 4.88±0.22 | 4.28±0.25 |
| 95.5±5.6 | 75.4±2.8* | 99.0±3.1 | 76.5±3.3* | 98.0±2.9 | 74.5±3.1* |
| 1.06±0.08 | 0.82±0.07* | 0.99±0.07 | 0.84±0.07* | 1.02±0.08 | 0.72±0.09* |
| 1.25±0.01 | 1.19±0.01* | 1.28±0.02 | 1.21±0.01* | 1.22±0.01 | 1.15±0.02* |
| 26.2±1.3 | 23.8±1.2* | 26.4±1.5 | 24.0±1.2* | 26.5±1.3 | 22.9±1.2* |
| 1.2±0.10 | 0.9±0.11* | 1.21±0.11 | 0.98±0.10* | 1.30±0.12 | 0.94±0.11* |
| 2.9±0.08 | 2.5±0.10* | 2.98±0.09 | 2.61±0.12* | 3.01±0.14 | 2.68±0.14* |

UDVA CDVA

Cylindrical refraction

Spherical refraction Spherical equivalent, D Keratometry of the anterior surface, a steep axis, D Keratometry of the posterior surface, a steep axis, D Thickness of cornea, apex, µm Volume of cornea, mm^3^

Index of asphericity (Q) Index of progression Astigmatism Index of surface variance (ISV) Index of vertical asymmetry (IVA) Keratoconus index (KI)

Index of height asymmetry (IHA) Surface regularity index (SRI) Surface asymmetry index (SAI)

CXL = corneal collagen cross-linking; ICRS = intrastromal corneal ring segments; UDVA = uncorrected distance visual acuity; CDVA = corrected distance visual acuity.

^*^P < .05 (comparison with preoperative data in all groups). ^P1 < .05 (comparison of postoperative data between ICRS + CXL and CXL; ICRS + CXL and ICRS groups)

125 125
126 126

**Table 2. Clinical parameters before and 6 months after TG-PRK in patients underwent CXL, ICRS or combined treatment**

Indices	CXL + TG-PRK	ICRS + TG-PRK	ICRS + CXL + TG-PRK

| before | after | before | after | before | after |
| --- | --- | --- | --- | --- | --- |
| 0.42±0.04 | 0.58±0.03* | 0,40±0,03 | 0,60±0,03* | 0.55±0.04 | 0.70±0.04*^ |
| 0.80±0.04 | 0.95±0.04* | 0,79±0,05 | 0,94±0,04* | 0.83±0.03 | 0.98±0.03* |
| -2.00±0.13 | -0.89±0.11 | -2,96±0,14 | -1,10±0,11 | -2.84±0.14 | -0.80±0.12 *^ |
| -1.89±0.10 | -0.68±0.09 | -1,88±0,10 | -0,64±0,11 | -1.87±0.10 | -0.51±0.09* |
| 5.10±0.11 | 4.89±0.11 | 5,18±0,14 | 4,84±0,10* | 5.08±0.12 | 4.12±0.11*^ |
| 48.31±0.12 | 46.10±0.09 | 44,32±0,11 | 44,11±0,08 | 50.21±0.12 | 42.07±0.09 |
| -7.78±0.07 | -7.88±0.06 | -7,80±0,07 | -7,86±0,06 | -7.80±0.07 | -7.84±0.05 |
| 448±3.8 | 380.1±3.0 | 445,0±4,2 | 376,0±3,1 | 446.0±4.0 | 370±3.0 * |
| 56.1±0.3 | 56.0±0.2* | 55,8±0,2 | 55,1±0,2* | 55.4±0.2 | 54.8±0.1* |
| -0.89±0.06 | -0.89±0.05 | -0,88±0,04 | -0,89±0,04 | 0.88±0.04 | 0.89±0.05 |
| 2.49±0.06 | 2.58±0.05 | 2,50±0,06 | 2,64±0,07* | 2.51±0.07 | 2.68±0.07* |
| 4.34±0.21 | 4.24±0.15 | 76,5±3,3 | 66,4±2,5* | 4.28±0.25 | 4.05±0.12 |
| 75.4±2.8 | 65.1±1.9* | 0,84±0,07 | 0,69±0,04* | 74.5±3.1 | 62.0±2.5* |
| 0.82±0.07 | 0.68±0.05* | 1,21±0,01 | 1,13±0,02* | 0.72±0.09 | 0.51±0.05*^ |
| 1.19±0.01 | 1.11±0.01* | 24,0±1,5 | 20,6±1,2* | 1.15±0.02 | 1.09±0.01* |
| 23.8±1.2 | 20.1±1.1* | 0,98±0,10 | 0,75±0,11 | 22.9±1.2 | 18.15±0.9* |
| 0.90±0.11 | 0.72±0.10* | 2,61±0,12 | 2,11±0,12* | 0.94±0.11 | 0.64±0.12* |
| 2.5±0.10 | 2.0±0.11* | 0,40±0,03 | 0,60±0,03* | 2.68±0.14 | 1.8±0.12* |

UDVA CDVA

Cylindrical refraction Spherical refraction Spherical equivalent, D

Keratometry of the anterior surface, a steep axis, D Keratometry of the posterior surface, a steep axis, D Thickness of cornea, apex, µm

Volume of cornea, mm^3^

Index of asphericity (Q)

Index of progression Astigmatism

Index of surface variance (ISV) Index of vertical asymmetry (IVA) Keratoconus index (KI)

Index of height asymmetry (IHA) Surface regularity index (SRI) Surface asymmetry index (SAI)

CXL = corneal collagen cross-linking; ICRS = intrastromal corneal ring segments;

TG-PRK = topography-guided photorefractive keratectomy; UDVA = uncorrected distance visual acuity; CDVA = corrected distance visual acuity.

^*^P < .05 (comparison with preoperative data in all groups). ^P1 < .05 (comparison of postoperative data between ICRS + CXL + TG-PRK and CXL + TG-PRK; ICRS + CXL + TG-PRK and ICRS + TG-PRK

groups)

127 127
128 These data confirm significant improvement of visual acuity and key topographic indices of
129 cornea after TG-PRK in all studied groups (Fig 2, Fig 3 and Fig 4). According to some
130 parameters (UDVA, cylindrical refraction and spherical equivalent, as well as index of vertical
131 asymmetry (IVA)) results were statistically significantly better in patients underwent previous
132 combination of CXL and ICRS.

# 133 Fig 2. Initial corneal topography in patient planned for ICRS+CXL+TG-PRK.

134 ICRS = intrastromal corneal ring segments; CXL = corneal collagen cross-linking; TG-PRK =
135 topography-guided photorefractive keratectomy.
136 **Fig 3. Corneal topography after ICRS+CXL.** ICRS = intrastromal corneal ring segments;
137 CXL = corneal collagen cross-linking.
138 **Fig 4. Corneal topography after ICRS+CXL+TG-PRK.** ICRS = intrastromal corneal ring
139 segments; CXL = corneal collagen cross-linking; TG-PRK = topography-guided photorefractive
140 keratectomy.

## 141 DISCUSSION

142 Researches from Dagestan Centre of Eye Microsurgery demonstrated high clinical efficacy of
143 combination of laser intrastromal keratomileusis with CXL, comparing to standard cross-
144 linking [9]. A huge number of published works are devoted to the study of the efficacy of
145 combined methods in the treatment of patients with keratoconus [1, 2, 3]. These works present
146 mainly the results of combined treatment, and there are just a few studies comparing the
147 efficacy of various types of combined treatment [6, 9].
148 Egyptian researchers presented the comparative results of two types of combined surgical
149 treatment in keratoconus: a combination of intra-tunnel CXL with implantation of ICRS and a
150 combination of epithelial CXL with ICRS [10]. There is a report on comparison of results after
151 CXL with results after its combination with ICRS [11]. The main difference between our work
152 and published data is that we compared not only the results of a combination of two (CXL and
153 ICRS) keratoconus treatment methods with non-combined options, but also different types of
154 two-stage surgical treatment in keratoconus using transepithelial TG-PRK. At the same time,
155 the higher efficacy of the staged use of the combined treatment was showed: CXL + ICRS as
156 the first stage and TG-PRK as the second stage.
157 Thus, combined two-stage (with 8-month interval) surgical treatment in patients with
158 keratoconus, consisting of implantation of ICRS with CXL followed by TG-PRK, is more
159 159
160 160
161 161
162 162
163 163
164 164
165 165
166 166
167 167
168 168
169 169
170 170
171 171
172 172
173 173
174 174
175 175
176 176
177 177
178 178
179 179

effective than the use of non-combined two-stage methods.

# 180 REFERENCES

181 1. Andreanos KD, Hashemi K, Petrelli M, Droutsas K, Georgalas I, Kymionis GD.
182 Keratoconus Treatment Algorithm. Ophthalmol Ther. 2017;6(2):245-262.
183 2. Iqbal M, Elmassry A, Tawfik A, Elgharieb ME, El Deen Al Nahrawy OM, Soliman
184 AH, et al. Evaluation of the Effectiveness of Cross-Linking Combined with
185 Photorefractive Keratectomy for Treatment of Keratoconus. Cornea. 2018;37(9):1143-

186	1150.

187 3. Karmel M, Hersh PS, Lin DT, et al. Keratoconus: consider combination therapy.
188 Clinical	update.	Cornea.	2013;31-33.	Available	from:

189	[https://www.aao.org/assets/dede14a6-4e5f-4335-8009-c99da14027d4/635570315230](https://www.aao.org/assets/dede14a6-4e5f-4335-8009-c99da14027d4/635570315230%20230000/march-2013-clinical-update-cornea-pdf)

190 [230000/march-2013-clinical-update-cornea-pdf](https://www.aao.org/assets/dede14a6-4e5f-4335-8009-c99da14027d4/635570315230%20230000/march-2013-clinical-update-cornea-pdf)
191 4. Arnalich-Montiel F, Alió Del Barrio JL, Alió JL. Corneal surgery in keratoconus:
192 which type, which technique, which outcomes? Eye Vis. 2016;3:2–14.
193 5. Al-Mohaimeed MM. Combined corneal CXL and photorefractive keratectomy for
194 treatment of keratoconus: a review. Int J Ophthalmol. 2019;12(12):1929-1938.
195 6. Pershin K.B., Gurmizov E.P., Pashinova N.F. i dr. Opyt kombiniro-vannogo lechenija
196 keratokonusa	(femtolazernaja	implantacija	intras-tromal'nyh	segmentov	s
197 posledujushhim	krosslinkingom	rogovichnogo	kollagena)	u	detej.	Rossijskaja
198 pediatricheskaja oftal'mologija. 2017;12(4):200-203. Russian.
199 7. Zhu AY, Jun AS, Soiberman US. Combined Protocols for Corneal Collagen Cross-
200 Linking with Photorefractive Surgery for Refractive Management of Keratoconus:
201 Update on Techniques and Review of Literature. Ophthalmol Ther. 2019 Oct;8(Suppl

202	1):15-31.

203 8. Stenton G. Mediko-biologicheskaja statistika. Moskva. Izd. Praktika. 1999;459.
204 Russian.
205 9. Aliev A-G.D., Aliev A.A-G., Magomedova A.B. Materialy Vserossijskoj kon-ferencii
206 oftal'mologov s mezhdunarodnym uchastiem. Chenak-kala. 2017; 36-40. Russian.
207 10. Hosny M, Nour M, Azzam S, Salem M, El-Mayah E. Simultaneous intratunnel cross-
208 linking with intrastromal corneal ring segment implantation versus simultaneous
209 epithelium-off cross-linking with intrastromal corneal ring segment implantation for
210 keratoconus management. Clin Ophthalmol. 2018;12:147-152.
211 11. Sharma IP, Bakshi R, Chaudhry M. Corneal collagen cross-linking with and without
212 simultaneous intrastromal corneal ring segment implantation: One-year pilot study. Eur
213 213
214 214
215 215
216 216
217 217
218 218
219 219
220 220
221 221
222 222
223 223

J Ophthalmol. 2021;31(1):61-68.

Figure 1	[Click here to access/download;Figure;Fig1.tiff](https://www.editorialmanager.com/pone/download.aspx?id=29920603&guid=34012566-e515-4d9f-8668-381771e12c27&scheme=1)

Figure 2	[Click here to access/download;Figure;Fig2.tif](https://www.editorialmanager.com/pone/download.aspx?id=29935910&guid=3d8c1cb5-a12d-4e7d-824f-066a33e94957&scheme=1)

Figure 3	[Click here to access/download;Figure;Fig3.tif](https://www.editorialmanager.com/pone/download.aspx?id=29935911&guid=53b1c6d1-29bd-46d5-9c3f-99e668ef7469&scheme=1)

Figure 4	[Click here to access/download;Figure;Fig4.tif](https://www.editorialmanager.com/pone/download.aspx?id=29935912&guid=397fa52e-050f-47b2-91b9-04892d6d08c7&scheme=1)
